# A Multicenter Phase II Rater-Blinded Randomized Controlled Trial to Compare the Effectiveness of Eye Movement Desensitization Reprocessing Therapy vs. Treatment as Usual in Patients With Substance Use Disorder and History of Psychological Trauma: A Study Design and Protocol

**DOI:** 10.3389/fpsyt.2019.00108

**Published:** 2019-03-15

**Authors:** Alicia Valiente-Gómez, Ana Moreno-Alcázar, Joaquim Radua, Bridget Hogg, Laura Blanco, W. Lupo, Víctor Pérez, Maria Robles-Martínez, Marta Torrens, Benedikt L. Amann

**Affiliations:** ^1^Centre Fòrum Research Unit, Institut de Neuropsiquiatria i Addiccions, Barcelona, Spain; ^2^IMIM (Hospital del Mar Medical Research Institute), Barcelona, Spain; ^3^Centro de Investigación Biomédica en Red de Salud Mental, Madrid, Spain; ^4^Institut de Neuropsiquiatria i Addiccions, Hospital del Mar, Barcelona, Spain; ^5^Department of Psychiatry, Universitat Autònoma de Barcelona, Barcelona, Spain; ^6^Institut d'Investigacions Biomèdiques August Pi i Sunyer, Barcelona, Spain; ^7^Department of Psychosis Studies, Institute of Psychiatry, Psychology & Neuroscience, King's College London, London, United Kingdom; ^8^Department of Clinical Neuroscience, Centre for Psychiatry Research, Karolinska Institutet, Stockholm, Sweden; ^9^Benito Menni Complex Assistencial en Salut Mental, Sant Boi de Llobregat, Spain; ^10^Department of Personality, Evaluation and Psychological Treatments, University of Barcelona, Barcelona, Spain; ^11^RETICS-Redes Temáticas de Investigación Cooperativa en Salud en Trastornos Adictivos, Barcelona, Spain

**Keywords:** substance use disorder, addiction, psychological trauma, comorbidity, EMDR therapy, treatment as usual

## Abstract

**Background:** Psychological trauma has a strong negative impact on the onset, course and prognosis of substance use disorders (SUD). Few trauma-oriented treatment approaches have been trialed, but preliminary evidence exists of the efficacy of Eye Movement Desensitization and Reprocessing (EMDR) therapy in improving clinical symptoms in SUD patients.

**Objective:** To assess if EMDR therapy leads to: (1) reduced substance consumption; (2) an improvement in psychopathological and in trauma-related symptoms; and (3) an improvement in overall functioning. Our hypothesis is that the EMDR group will improve in all variables when compared to the treatment as usual (TAU) group at 6 and 12-months visits.

**Method:** In this multicenter phase II rater-blinded randomized controlled trial, 142 SUD patients with a history of psychological trauma will be randomly assigned to EMDR (*n* = 71) or to TAU (*n* = 71). Patients in the EMDR group will receive 20 psychotherapeutic sessions of 60 min over 6 months. Substance use will be measured using the Timeline Followback Questionnaire, the Dependence Severity Scale and the Visual Analog Scale. Traumatic events will be measured by The Holmes-Rahe Life Stress Inventory, the Childhood Trauma Questionnaire Scale, the Global Assessment of Posttraumatic Stress Questionnaire, the Impact of Event Scale-Revised and the Dissociative Experiences Scale. Clinical symptomatology will be evaluated using the Hamilton Depression Rating Scale, the Young Mania Rating Scale and the Brief Psychiatric Rating Scale. Functionality will be assessed with the Functioning Assessment Short Test. All variables will be measured at baseline, post-treatment and 12 months as follow-up. Primary outcome: to test the efficacy of EMDR therapy in reducing the severity of substance use. The secondary outcomes: to test the efficacy in reducing trauma-related psychological symptoms and psychopathological symptoms and in improving overall functioning in patients with comorbid SUD and a history of psychological trauma.

**Conclusion:** This study will provide evidence of whether EMDR therapy is effective in reducing addiction-related, trauma and clinical symptoms and in improving functionality in patients with SUD and a history of trauma.

**Clinical Trial Registration:** The trial is registered at ClinicalTrials.gov, identifier: NCT03517592.

## Introduction

Substance use disorders (SUD) represent an important social and public health problem due to their negative consequences in terms of delinquency, family disintegration, academic and occupational disengagement, mental illness, transmission of infectious diseases, intoxication, and mortality rates (1). One significant risk factor for problematic alcohol and substance use is the presence of multiple adverse childhood events, as shown by a recent meta-analysis in the Lancet (2). Different studies have found that between 66 and 97.4% of SUD patients have experienced Criterion A traumatic events according to DSM IV criteria (3–7), and the prevalence of post-traumatic stress disorder (PTSD) in inpatients with SUD is estimated to range from 25–51%, two to four times more than general population rates ranging from 1.3 to 12.3% (8). Even adverse events not meeting PTSD Criterion A have an important impact in the course of the disorder because they have been shown to be associated with substance use outcomes (2).

In order to clarify the difference between psychological trauma, PTSD and subthreshold PTSD, we will provide a brief definition of them. Psychological trauma is a term that refers to any life event that causes discomfort to the subject and exceeds an individual's abilities to integrate the emotions involved with the experience (9). The term PTSD refers to, based on DSM-V criteria (10), exposure to actual or threatened death, serious injury, or sexual violence, accompanied by the presence of the following trauma-related symptoms: intrusion, persistent avoidance of stimuli, negative alterations in cognitions and mood and marked alterations in arousal and reactivity. Symptoms must have a duration of more than 1 month and cause clinically significant distress or impairment in the patient's overall functioning, and not be attributable to the physiological effects of a substance or another medical condition (10). Finally, the term subthreshold PTSD should be used when the clinical presentation does not meet all criteria for PTSD diagnosis.

A large study into populations addicted to alcohol and various illicit drugs found that PTSD and subsyndromal PTSD were correlated with addiction severity, a worse disease prognosis, more hospital admissions, poorer response to treatment, shorter periods of abstinence and greater craving (4). These characteristics make this specific population difficult to treat. In addition, trauma is highly prevalent in patients with severe mental disorders (SMDs) such as depression, bipolar disorder or psychosis, where PTSD and SUD are frequently comorbid and negatively influence the course of mental illness (11, 12). In some cases, disease course is worsened by a high prevalence of trauma-related dissociative symptoms (13). These findings suggest psychological trauma should be assessed in SUD and in dual pathology patients and be included as an objective of the treatment plan (12, 14).

Along these lines, even though pharmacological treatment such as selective serotonin reuptake inhibitors, atypical antipsychotics and benzodiazepines have demonstrated a limited efficacy in the treatment of PTSD and psychological trauma, some drugs such as prazosin could be a promising strategy to consider in adjuvant treatment with the psychological approach, given the positive results shown in specific PTSD symptoms as reported the meta-analysis of Berardis et al. (15).

A recent Cochrane review into the efficacy of CBT-based therapies for comorbid SUD and PTSD found that preliminary evidence showed that trauma-focused therapy delivered alongside SUD therapy could improve PTSD symptoms at short- and long-term, and SUD symptoms at long-term, compared to treatment as usual. They concluded that more research is needed on trauma-focused therapies for this population, but that there is very little evidence to support individual therapies not focused on trauma (16).

One leading trauma-focused treatment is Eye Movement Desensitization and Reprocessing (EMDR) therapy, recommended as a first-line PTSD treatment by international bodies such as the American Psychiatric Association (APA) (17) and the World Health Organization (WHO) (18). Initial studies have shown the potential for EMDR to be applied to the SUD population. Until nowadays, there are three published trials in SUD and EMDR: two small randomized controlled trials (RCT) and one non-randomized controlled trial of EMDR vs. treatment as usual (TAU) in patients with SUD (19–21). They have suggested that EMDR, compared to the control group, significantly improves craving (19), depression, anxiety, self-esteem (19, 20), and dissociative symptoms (21), but results must be repeated with larger samples. A large scale RCT is currently underway (22) to determine the efficacy of EMDR in reducing PTSD symptoms in an inpatient sample with comorbid SUD and PTSD or sub-threshold PTSD.

In the current study, we aim to carry out the first large RCT into the impact of EMDR on SUD symptoms in outpatients (with or without comorbid SMDs) with a history of psychological trauma. This is groundbreaking in, firstly, focusing on addiction outcomes instead of PTSD symptoms as a primary outcome and, secondly, in including all patients showing trauma-associated symptoms, even if they do not fulfill PTSD or subsyndromal PTSD criteria.

Additionally, EMDR is an interesting psychotherapeutic tool for this population due to its potential to improve the course of comorbid SMDs, where strong preliminary evidence also shows EMDR to be a promising treatment beyond PTSD (23), such as in bipolar disorder (24), or psychosis (25), as well as in depression (26, 27) and anxiety (28–30). The evidence that EMDR is efficacious in these kind of patients will help facilitate its application in complex real-world settings.

## Methods

This is a multicenter phase II rater-blinded randomized controlled trial, phase II, with two parallel branches, EMDR and TAU, of patients diagnosed with SUD who have a comorbid history of psychological trauma, even if they do not currently meet DSM-V criteria for PTSD. The patients will be matched by site, age, sex, and diagnosis. One group will consist of TAU plus 20 individual 60-min EMDR sessions over a duration of 6 months, while the other group will receive TAU only. Patients will be evaluated at baseline (T0), 3 months (T1: only substance use-related symptoms), post-treatment at 6 months (T2), and at 12 months as follow-up (T3) (see Table 1). Clinical raters carrying out evaluations will be blind to the participants' research condition. Patients will not be blind to treatment as a sham alternative to EMDR therapy is impossible due to its use of bilateral stimulation.

**Table 1:** SPIRIT flow diagram: schedule of enrolment, interventions, and assessments.

	**Study period**				
	**Enrolment**	**Allocation**	**Post-allocation**		**Close-out**
**TIMEPOINT**	***–t_**1**_ & t_**0**_***	**0**	***t_**1**_***	***t_**2**_***	***T_**3**_***
**ENROLMENT**					
Eligibility screen	X				
PRISM	X				
MINI	X				
HDRS	X			X	X
YMRS	X			X	X
BPRS	X			X	X
TLFB	X		X	X	X
SDS	X		X	X	X
EVA	X		X	X	X
CTQ	X				
H-RLSI	X				
EGEP	X			X	X
IES-R	X			X	X
DES	X			X	X
Informed consent	X				
Allocation		X			
**INTERVENTIONS**					
EMDR					
TAU					
**ASSESSMENTS**					
FAST	X			X	X

*PRISM, Psychiatric Research Interview for Substance and Mental Disorders; MINI, Mini-International Neuropsychiatric Interview; HDRS, Hamilton Depression Rating Scale; YMRS, Young's scale for the evaluation of the Mania; BPRS, Brief Scale of Psychiatric Evaluation; TLFB, Timeline Followback; SDS, Dependence severity scale; EVA, Analog visual scale; CTQ, Childhood Trauma Questionnaire; H-RLSI, The Holmes-Rahe Life Stress Inventory; EGEP, Global Assessment of Posttraumatic Stress Questionnaire; IES-R, Scale of the impact of events reviewed; DES, Scale of Dissociative Experiences; EMDR, Eye movement desensitization and reprocessing therapy; TAU, Treatment as usual; FAST, Functioning Assessment Short Test*.

The study has been approved by the Ethic Committees of the Hospital Benito Menni, Germanes Hospitalàries del Sagrat Cor de Jesús (PR-2018-04), and the IMIM, Parc de Salut Mar (2017/7615/I). All participants will sign informed consent prior to enrollment. Details of the trial design can be also gathered from Supplementary Material (Standard Protocol Items: Recommendations for Interventional Trials [SPIRIT] Checklist).

This is a multicenter phase II collaborative project will involve the participation of five different centers from the Barcelona catchment area, Spain: four outpatient addiction clinics pertaining to the Institute of Neuropsychiatry and Addictions (INAD), Parc de Salut Mar, and a fifth pertaining to the Hospital Benito Menni, in Sant Boi de Llobregat. Both institutions involved are centers of reference in mental health treatment and research, facilitating the recruitment and development of the project. EMDR therapists have extensive experience in EMDR protocols and SUD. All participating raters will be trained in the blind-to-treatment application of all clinical assessments.

### Study Outcomes

The primary outcome is to test the efficacy of EMDR therapy in reducing the severity of substance use in patients with comorbid SUD and a history of psychological trauma, irrespective of whether the patient meets DSM-V PTSD criteria. Changes from baseline in the severity of substance use are measured by the Timeline Follow Back (TLFB) (31), the Severity of Dependence Scale (SDS) (32), and the Visual Analog Scale (VAS).

The secondary outcome is to test the efficacy of EMDR in improving trauma-related psychological symptoms, psychopathological symptoms, and the overall functionality in SUD patients with a history of psychological trauma. Changes in psychological trauma and dissociative symptoms are measured using the Global Assessment of Posttraumatic Stress Questionnaire (EGEP-5) (33), the Impact of Event Scale-Revised (IES-R) (34), and the Dissociative Experiences Scale (DES) (35), respectively. The type and severity of traumatic experiences are measured using the Childhood Trauma Questionnaire (CTQ) (36) and The Holmes-Rahe Life Stress Inventory (37). Changes in psychopathological symptoms are measured by the Hamilton Depression Rating Scale (HDRS) (38), Young Mania Rating Scale (YMRS) (39), and the Brief Psychiatric Rating Scale (BPRS) (40). Changes in overall functioning will be measured using the Functioning Assessment Short Test (FAST) (41).

### Hypotheses

1. Patients in the EMDR group will show a reduction in the level and severity of substance use-related symptoms as compared to the TAU group.
2. Patients in the EMDR group will show a reduction in the number of relapses as compared to the TAU group.
3. Patients in the EMDR group will show a reduction in the severity of trauma-related symptoms as compared to the TAU group.
4. There will be a reduction in depressive symptoms associated with a comorbid psychiatric disorder in the EMDR group as compared to the TAU group.
5. There will be a reduction in (hypo) manic symptoms associated with a comorbid psychiatric disorder in the EMDR group as compared to the TAU group.
6. There will be a reduction in general psychopathology symptoms associated with psychiatric comorbidity in the EMDR group as compared to the TAU group.
7. Patients in the EMDR group will show an improvement in functioning as compared to the TAU group.

### Study Participants

The study sample will consist of 142 outpatients fulfilling the criteria of the Diagnostic and Statistical Manual of Mental Disorders, fifth edition (DSM-5), for SUD based on a clinical interview (Psychiatric Research Interview for Substance and Mental Disorders; PRISM) (42) and a review of case notes.

Inclusion criteria are: (1) aged 18–65, (2) outpatient, and (3) presence of one or more traumatic events currently causing trauma-associated symptoms (Impact of Events Scale-Revised, IES-R > 0) and Subjective Disturbance Unit (SUD) > 5, that assesses subjective disturbance in a scale between 0 and 10, but it is not necessary that traumatic events meet DSM-5 criteria for PTSD.

Exclusion criteria are: (1) presence of organic brain diseases, (2) presence of acute suicidal ideation, (3) having received a trauma-focused therapy or attended psychotherapeutic groups for survivors of violence within the last 2 years, or (4) acute episode of comorbid psychiatric disorder.

### Randomization Procedure

Clinical trials use randomization to balance confounding factors (to an uncontrolled extent) and to conceal allocation. However, complete randomization in small to moderate studies may fail to balance groups, severely affecting inference. To overcome this issue Efron (43) introduced the biased coin methods, which randomize each patient to one or the other group with a probability or another with the aim of increasing the balance of known confounding factors. Importantly, these methods still randomize each of the patients, thus balancing the unknown confounding factors (to the uncontrolled extend that complete randomization does) and concealing allocation (44). Non-deterministic dynamic allocation designs, such as the biased coin methods, were included in international guidelines for drug clinical trials (45) adopted by the European Community, Japan, United States FDA, Canada and Switzerland (Implementation of E9 Statistical Principles for Clinical Trials. URL: https://www.ich.org/products/guidelines/efficacy/efficacy-single/article/statistical-principles-for-clinical-trials.html).

In this trial, all patients meeting the inclusion criteria will receive the baseline (T0) assessment. After T0, participants will be assigned to the EMDR or TAU group following a biased coin procedure (46): (1) the first two patients will be randomly allocated to EMDR with *p* = 0.5, (2) the next patient will be allocated as follows: (b1) if one group already includes at least two more patients than the other group, the patient will be randomly allocated to EMDR with *p* = 0.8 is this is the smallest group and with *p* = 0.2 if it is the largest group, (b2) otherwise, we will first simulate that the patient is allocated to EMDR and calculate the sum of the between-group square standardized differences in site, age, sex, diagnosis (dual vs. not dual) and number of substances consumed during the month before the randomization (none. one or more than one), we will then simulate that the patient is allocated to TAU and recalculate the sum, and finally randomly allocate the patient to EMDR with *p* = 0.8 if this was associated to the smallest sum and with *p* = 0.2 if not. For example, if we had already included 10 patients to the EMDR group and 8 patients to the TAU group, the 19th patient would be randomly allocated with *p* = 0.2 for EMDR and *p* = 0.8 for TAU. If he/she was allocated to TAU, for the 20th patient we would calculate the above sum of covariates after simulating that the he/she is allocated to EMDR and after simulating that he/she is allocated to TAU, and if the sum of the EMDR simulation was larger than the sum of the TAU simulation, we would randomly allocate the 20th patient with *p* = 0.2 for EMDR and *p* = 0.8 for TAU. Following this procedure, the final groups should be balanced in size and matched in site, age, sex and diagnosis. All steps of the randomization process will be automatically carried out by an independent researcher in a central location using a computer program.

### Computation of Sample Size

The study aims to assess the efficacy of EMDR therapy compared with TAU, in inpatients with SUD, in terms of a reduction in substance use, a reduction in symptoms associated with craving and associated symptoms of anxiety and depression, and an improvement in functioning.

We aim for the study to be able to detect medium-sized differences in the pre-post changes between groups with an 80% statistical power. Given that there are no previous studies that report the variability of these changes, we have defined medium-sized differences as those with a medium effect size (*d* = 0.5). With the function power.t.test from R (http://www.r-project.org/), we have calculated that the number of patients required to detect medium-sized differences (*d* = 0.5) with a statistical power of 80% is *n* = 64 per intervention group (two groups, total *n* = 128). Assuming a loss percentage of approximately 10% of the patients in the study, it would be necessary to recruit approximately 142 patients, 71 for each intervention branch.

### Statistical Analysis

The distribution of socio-demographic and clinical characteristics between groups in the baseline state will be summarized using descriptive statistics. The change in clinical and functional variables with regard to baseline evaluation at strategic points of the intervention will be analyzed using mixed-effects repeated-measures linear models, including as fixed factors time, treatment conditions and their interactions, and as a random factor the site. The differences between groups, for the categorical variables and main clinics, will be analyzed by adding covariates to the models. Those covariates that are statistically significant may be added in the same model to determine which covariates are best predictors of the response. The size of the effects will be estimated using the g of Hedges or the r of Pearson. It will be corrected for multiple comparisons. The statistical software used for the analysis will be the latest available version of R. We will conduct an intention to treat (ITT) analysis. The “Last Observation Carried Forward” (LOCF) method will be used for losses of follow-up.

## Stepwise Procedures

### Intervention

#### EMDR

Patients in the EMDR condition will receive 20 individual sessions of 60 min each, using the standard EMDR therapy protocol developed by Shapiro (47) and a further specific protocol for SUD, the CRAVEX protocol, developed by Hase (48) to treat both trauma-related symptoms and SUD symptoms, respectively.

The current standard protocol includes eight phases, briefly described below:

1. Patient history: The therapist assesses the patient's attachment history, medical history, physical problems and identifies traumatic events and their relationship to current symptoms. A treatment plan is developed.
2. Patient preparation: A safe therapeutic environment is established, the theory and processes of EMDR are explained, and the therapist may try out different modalities of bilateral stimulation, including eye movements, where the patient's eyes follow the therapist's fingers moving in horizontally or diagonally across their field of vision. While eye movements are generally recommended, if they are not well-tolerated, another modality, such as tapping the back of the patient's hands or auditory tones, may be used.
3. Patient assessment: The therapist helps the patient to bring the traumatic memory to mind and identify associated cognitions, emotions and physical sensations. The patient identifies the image which represents the worst part of the traumatic memory and an associated negative cognition, and is helped to identify a positive cognition to replace this. Finally, the patient identifies the distress level they experience upon bringing the traumatic experience and negative cognition to mind. Distress is measured using the Subjective Units of Disturbance Scale (SUD), scored from 0 (minimum disturbance) to 10 (maximum disturbance).
4. Memory desensitization: The patient brings to mind the traumatic image, negative cognition and associated emotion and notices any physical discomfort generated in the present moment. The patient focuses on this material and a 30–40 s set of bilateral stimulation is applied, during which the patient is instructed to observe what is happening without judgment, and afterwards express what occurred. The therapist asks the patient to focus on new material without comment, assessment, or interpretation. This is repeated until no new material arises and the traumatic memory generates no distress (SUD = 0 or 1).
5. Installing the positive cognition: The patient brings the positive cognition and original experience to mind. Further sets of bilateral stimulation are applied, causing the patient to link the positive cognition with the original memory, until the positive belief is fully installed.
6. Body scan: The therapist asks the patient to close their eyes and focus on the original experience and positive cognition, and to notice if any sensation arises. If a negative or uncomfortable sensation is reported, the therapist will resume bilateral stimulation until it disappears. If the sensation is positive, it will be reinforced with short sets (10–12 s) of bilateral stimulation.
7. Closure: The therapist explains possible effects following the session, such as new insights, thoughts, memories, and even dreams or nightmares, and offers recommendations about what to do in each case.
8. Reevaluation: The therapist assesses the patient's experiences since the previous session and reevaluates the traumatic memory to confirm functional processing. If the memory has been desensitized, the therapist selects a new target of either another traumatic memory, a current trigger of distress, or a potentially threatening future event.

The CRAVEX protocol (48) will also be applied to process craving. This protocol focuses on the concept of addiction memory (AM), in which the biological effect of the drugs has a serious impact on the brain comparable to trauma, which leads to the formation of a maladaptive implicit memory which if reprocessed can decrease cravings or urges, and potentially promote access to the brain channels connected to the initial reasons the individual became addicted (49). Instead of SUD, the CRAVEX protocol uses Level of Urge (LOU), with a scale of 0 (no urge) to 10 (worst urge imaginable), referring to the urge to consume the substance. As in the standard protocol, positive and negative cognitions, emotions and physical sensations are identified but, in this case, they are associated with the last time that the subject consumed the drug. Bilateral stimulation is used until the AM is desensitized, and finally the positive cognition is installed.

#### TAU

All patients included in this study will participate in the TAU condition, comprising the standard care package offered by the Drug and Alcohol Outpatient Unit. This is a multidisciplinary unit with doctors, psychiatrists, clinical psychologists, nurses, and social workers on the staff. An individual care plan is drawn up depending on individual needs and may include follow-up psychiatric visits to evaluate clinical status and, if necessary, readjust pharmacological treatment, and psychological visits to assess and detect risk situations and prevent relapses using a non-trauma focused CBT. In no case will psychological treatment focus on PTSD. TAU also includes nurse visits for health and self-care habits and nurses may also carry out abstinence controls.

### Dropouts and Follow-Up

If a participant requires an inpatient stay due to substance misuse or an acute episode of a comorbid disorder during the 6-month intervention period, the patient will be excluded from the trial and considered as dropout because the hospital admission will mean the patient cannot continue with the EMDR psychotherapy during the acute phase. In the case of relapse during follow-up, patients will be maintained in the trial to obtain maximum information on the course of the illness.

## Materials and Equipment

### Instruments and Measures

The consumption of substances will be quantified using the following tools (see Table 2):

1. Timeline Followback Questionnaire (TLFB): The TLFB (31) is a retrospective calendar-based measure of daily substance use, initially developed to obtain self-reports of alcohol use but nowadays also used for other substances.
2. Severity of Dependence Scale (SDS): The SDS (32) is a 5-item questionnaire indicating the degree of dependence on different types of drugs. Each item is scored on a 4-point scale (0–3) and summed to create a total score. Higher scores indicate greater dependence.
3. Visual Analog Scale (VAS): A self-report scale to measure craving intensity, classically used to measure pain intensity. It ranges from 0 to 10: the higher the score, the greater the craving severity.

**Table 2:** Measurements to evaluate consumption symptoms.

**Clinical variable**	**Measurement interview/Self-report**	**T0**	**T1**	**T2**	**T3**
		**Baseline**	**Mid-treatment**	**Post-treatment**	**Post-treatment**
				**6 months**	**12 months**
Timeline	TLFB	x	x	x	x
Dependence	SDS	x	x	x	x
Craving	EVA	x	x	x	x

*TLFB, Timeline Followback Questionnaire; SDS, Severity of Dependence Scale; EVA, Visual Analog Scale*.

Trauma-related symptoms will be evaluated using the tools listed in Table 3:

1. Childhood Trauma Questionnaire (CTQ): The CTQ (36), Spanish validation (50), is a self-administered 28-item scale developed as a screening tool for histories of childhood abuse and neglect, with 5 subscales: emotional, physical or sexual abuse, and emotional or physical neglect. A 5-point Likert scale is used for the responses, ranging from “Never True” to “Very Often True.”
2. Global Assessment of Posttraumatic Stress Questionnaire(EGEP-5): The EGEP-5 (51) is a clinical interview for the diagnosis of PTSD, both current and in the past, based on DSM-V criteria. This scale contains three sections: events, symptoms and functioning.
3. The Holmes-Rahe Life Stress Inventory (37); Spanish validation (52): this is a scale assessing the frequency of 43 common stressful life events over the past 12 months, providing a standardized measure of their impact (53). Scores below 150 reflect low levels of stress, scores between 150 and 299 represent a 50% risk of a stress-related illness in the near future and scores above 300 represent an 80% risk (37), although each individual's reactions to stress and coping ability must be considered.
4. Impact of Event Scale-Revised (IES-R): The IES-R (54), Spanish validation (55), is a 22-item self-report measure of subjective distress over the previous 7 days related to a specific stressful life event. Items correspond directly to 14 of the 17 DSM-IV symptoms of PTSD. Items are rated on a 5-point scale ranging from 0 and 4, yielding a total score ranging from 0 to 88, with subscale scores for Intrusion, Avoidance, and Hyperarousal.
5. Dissociative Experiences Scale (DES): The DES (36), Spanish validation (56), is a 28-item self-reported questionnaire measuring a wide range of dissociative experiences, from normal to pathological, with an overall mean score ranging from 0 to 100 (57).

**Table 3:** Measurements to evaluate psychological trauma symptoms.

**Clinical variable**	**Measurement interview/Self-report**	**T0**	**T1**	**T2**	**T3**
		**Baseline**	**Mid-treatment**	**Post-treatment**	**Post-treatment**
				**6 months**	**12 months**
Childhood T	CTQ	x			
PTSD	EGEP	x		x	x
Traumatic events	H-RLSI	x			
Trauma's impact	IES-R	x		x	x
Dissociation	DES	x		x	x

*Childhood T, Childhood trauma; CTQ, Childhood Trauma Questionnaire; PTSD, post-traumatic stress disorder; EGEP, Global Assessment of Posttraumatic Stress Questionnaire; H-RLSI, The Holmes-Rahe Life Stress Inventory; IES-R, Impact Event Scale; DES, Scale of Dissociative Experiences*.

Diagnosis, clinical symptoms, and functioning will be assessed using the following instruments (see Table 4):

1. The clinical diagnosis of SUD will be made according to DSM-5 criteria, using the Spanish version of the clinical interview Psychiatric Research Interview for Substance and Mental Disorders (PRISM) (42).
2. Mini-International Neuropsychiatric Interview (MINI): A Spanish-validated brief structured diagnostic interview to assess the 17 most common psychiatric disorders as per DSM-IV criteria (58).
3. Hamilton Depression Rating Scale (HDRS): The HDRS (38), Spanish validation (59), is a 17-item hetero-administered scale designed to be used in patients previously diagnosed with depression, to quantitatively assess the severity of and changes in depressive symptoms. Each item has three or five possible answers, scored 0–2 or 0–4, respectively. Total scores range from 0 to 52.
4. Young Mania Rating Scale (YMRS): The YMRS (39), Spanish validation (60), is an 11-item hetero-administered scale which quantifies the severity of manic and hypomanic episodes. Four items are given more weight to compensate for poor cooperation from severely ill patients and are graded on a 0–8 scale (irritability, speech, thought content and disruptive/aggressive behavior), while the remaining seven items are graded on a 0–4 scale.
5. Brief Psychiatric Rating Scale (BPRS): The BPRS (40), Spanish validation (61), is an 18-item hetero-administered scale measuring psychopathological changes. It includes anxious, affective and psychotic symptoms, with each rated on a severity scale of 1–7.
6. Functioning Assessment Short Test (FAST): The FAST (41) is a 24-item instrument to evaluate functioning in six areas: autonomy, occupational functioning, cognitive functioning, finances, relationships and leisure. Each item is rated on a 4-point scale and summed to obtain a global score ranging from 0 to 72. The higher scores indicate poorer functional status.

**Table 4:** Measurements to evaluate clinical symptoms and functionality.

**Clinical variable**	**Measurement interview/Self-report**	**T0**	**T1**	**T2**	**T3**
		**Baseline**	**Mid-treatment**	**Post-treatment**	**Post-treatment**
				**6 months**	**12 months**
Diagnosis	PRISM	x			
Diagnosis	MINI	x			
Mania	YMRS	x		x	x
Depression	HDRS	x		x	x
Psychiatric S.	BPRS	x		x	x
Functionality	FAST	x		x	x

*PRISM, Psychiatric Research Interview for Substance and Mental Disorders; MINI, Mini-International Neuropsychiatric Interview; YMRS, Young Mania Rating Scale; HDRS, Hamilton Depression Rating Scale; Psychiatric S, Psychiatric symptoms; BPRS, Brief Scale of Psychiatric Evaluation, FAST, Functioning Assessment Short Test; Psychiatric S, Psychiatric symptoms*.

### Anticipated Results

The expected results of the current study will provide evidence of whether EMDR therapy is effective in reducing symptoms related to substance use, trauma-related and clinical symptoms in outpatients with SUD and a comorbid history of psychological trauma.

## Discussion

In recent years, the third-generation psychotherapies such as EMDR, which emerged in the 90s within the tradition of behavioral therapy (62), have gained considerable interest in both social and scientific fields due to their ability to integrate cognitive, emotional and behavioral components within the same psychotherapeutic approach, processing adverse life events and therefore ameliorating associated psychiatric symptoms. To date, preliminary evidence exists suggesting EMDR therapy is efficacious in SUD patients, thanks to previous studies done in that population (20, 63). In a more recent publication (21), a quasi-experimental study of 40 SUD outpatients found that EMDR as an add-on treatment had a pronounced effect in reducing post-traumatic and dissociative symptoms and also caused a significant improvement in the global severity of psychiatric symptoms. The aim of our trial is to provide further evidence of a positive effect of EMDR in this difficult-to-treat population in a larger RCT. In our study, we aim to treat real-world dual patients, with a wide range of substances used and varied comorbid psychiatric and somatic illnesses (except neurological disorders). In contrast to prior studies, we also include a year follow-up to test whether possible improvements are maintained. With results from a large RCT, we aim to promote trauma-oriented therapies in patients with dual disorders. Although it has been shown that there is a clear association between PTSD and addiction (64), most mental health care programs do not offer trauma-oriented therapies for patients with SUD. To understand better the relationship between the presence of traumatic events and the diagnosis of SUD, we will study several variables before, during and after treatment. We will assess variables of clinical severity, consumption and overall functioning with specific and validated instruments. In short, the potential of this study is to demonstrate the effectiveness and safety of EMDR in dual disorders. Along these lines, we could be closer to establishing an effective new psychological treatment for these patients in addition to the standard treatment. In this way, the impact of this trial is to improve the clinical evolution and prognosis and to reduce hospital admissions in SUD patients.

## Limitations

A limitation of this study is the inclusion of heterogeneous patients with various psychiatric diagnoses, including SUD with somatic and other psychiatric comorbidities. This trial has been specifically designed as a pragmatic real-world study with few exclusion criteria. We believe this limitation can be sufficiently controlled by matching samples in both arms and by the fact that the main common clinical underlying variable of all patients is a comorbid psychological trauma. Furthermore, the lack of control regarding drug treatment is a potential source of bias. To partly overcome this limitation, the “pharmacological treatment” variable will be taken into account and the treatment regimen should not be changed as far as possible, once patients have been stabilized. Moreover, it should be noted that our center has extensive experience in the treatment of SUD and uses standardized treatment protocols, which will also help limit this potential issue.

## Author Contributions

BA and MT had the idea for the project. AV-G, AM-A, LB, MR-M, and BA contributed to the design of the study. AV-G wrote the first draft of the manuscript, with supervision from AM-A, BH, and BA (primary supervisor). JR will carry out the randomization of patients and the statistical analyses. JR, LB, BH, WL, MR-M, VP, and MT contributed to the revisions and modifications of the manuscript and all have approved the final version.

### Conflict of Interest Statement

BA is the Research Committee Chair of EMDR Europe and he has been invited as speaker to various national and international congresses of EMDR. WL is supervisor of the Spanish Association of EMDR. The remaining authors declare that the research was conducted in the absence of any commercial or financial relationships that could be construed as a potential conflict of interest.
